# Mysophobia

**Published:** 1880-03

**Authors:** William A. Hammond

**Affiliations:** Professor of Diseases of the Mind and Nervous System in the Medical Department of the University of the City of New York, and in the Medical Department of the University of Vermont, etc.


The Practitioner.

VOL. I.—*March, 1880.*—NO. 3.

ARTICLE I.

### MYSOPHOBIA.

READ BEFORE THE NEW YORK NEUROLOGICAL SOCIETY,

BY WILLIAM A. HAMMOND, M. D.

(Professor of Diseases of the Mind and Nervous System in the Medical Department of the University of the City of New York, and in the Medical Department of the University of Vermont, etc.)

Under the name of Mysophobia (*Μύσος, defilement, pollution, contamination*, and *Φόβος, fear*), I propose to describe a form of mental derangement of which several cases have come under my charge, and which, I think, has not received that attention from psychiatric physicians which its importance deserves. As the name I have given it implies, it is characterized by a morbid, overpowering fear of defilement or contamination.

Under the designation of *La folie du doute* (*avec délire du toucher*) M. Le Grand du Saulle* has described an affection which has some marked relations with that which I now bring to the notice of the society, and cases in which this fear of contamination was a prominent feature, have been reported by other authors; but no one, previous to this time, has, so far as I know, called special attention to the remarkable phenomenon, which, I think, particularly characterizes the disorder, which is, in fact, the essential symptom, and which ought, I conceive, to determine its nomenclature. The instances which have come under my observation were not, with one exception, marked by any feeling of doubt in the minds of the subject, or necessarily with

* La folie du doute (avec délire du toucher), Paris, 1875.

derangement of the sense of touch, although, as in the cases cited by Le Grand du Saulle, there was more or less implication of this sense.

In all, ten cases constitute the basis of my experience with this form of mental disorder, two of which are now under my charge, one as a private patient, the other at my clinique at the University Medical College. Of the first seven cases my notes are not very complete. I was not particularly impressed with the fact of the distinctive character of the affection, though in all I find it stated that the subjects had delusions in regard to pollution, and were constantly washing the hands in order to remove taints, which, it was conceived, had been obtained by contact with various objects. It was not till the eighth case was observed, that I made thorough study of the existing phenomena, and recorded fully the symptoms that came under notice. I shall, therefore, limit my detailed descriptions to these last three cases, and then, after a few general remarks relative to the characteristics, pass to the consideration of the means which I have found most efficacious in the treatment.

CASE VIII.—M. G——, a lady thirty years of age, and a widow for three years, consulted me February 20, 1877, for what was considered to be incipient insanity, and an affection in all probability requiring, it was feared, incarceration in a lunatic asylum. The patient was quiet and orderly in her demeanor, and, so far as friends' accounts went, entirely sane, except on the one point of fear of contamination, which was exhibited by mental distress, and the practice of repeatedly washing her hands without there being obvious cause for so doing. She was perfectly coherent in regard to her clinical history, and I obtained from her the following account, which I transcribe in her own language.

"I was, about six months ago, reading a newspaper one evening, in which I came across an account of a man who, it was believed, had contracted small-pox from handling bank-notes which had been a short time previously in the possession of a person suffering from that disease. The circumstance made a deep impression on my mind, and, as I had only a few moments before counted quite a number of notes, the idea struck me that perhaps they had been handled by some person with a contagious disease of some kind or other. I had washed my hands just after counting these notes, but thinking that I had not possibly removed all the taint, I immediately washed them again. I went to bed feeling quite uncomfortable, and the next morning paid more than usual attention to the washing of my hands. I then recollected that I had placed the notes in a drawer of my dressing-table, in contact with linen which I had proposed putting on that day. I changed my intention, however, and selected some from another drawer instead, sending the other to the laundry. I then put on a pair of gloves, took out the notes, placed them in a letter-envelope, and had the drawer thoroughly washed with soap and water.

"Reflection upon the matter brought to mind the fact that after counting the notes I had touched various things before washing my hands. I could not recall what these were, and hence I was made very uncomfortable, for the idea occurred to me that I must have touched some of these things after washing my hands, and that, therefore, I was still in danger. The very dress that I wore then was the same that I have on now, and my hands had been more or less in contact with it all the morning. I felt myself accordingly forced to wash my hands, to take off the dress, and again to wash my hands.

"From that I went on from one thing to another. There was no end to the series. I washed everything I was in the habit of touching, and then washed my hands. Even the water was a medium for pollution. For no matter how thoroughly I wiped my hands after washing in it, a portion still remained, and this had to be washed off, and then again the hands washed. There was no end to it. The soap became associated in my mind with contamination, and I never used the same piece twice.

"Now, I can touch nothing without feeling irresistibly impelled to wash my hands afterward. If I am prevented doing so, I experience the most horrible sense of fear. I am always looking at my hands to ascertain if I can see anything on them, and I have a lens which I use to aid my eyesight. I have no particular apprehension of contracting small-pox, or any other disease that I can specify. It is an overpowering feeling that I shall be defiled in some mysterious way that presses on me with a force I cannot resist. As to shaking hands with any person, nothing would persuade me to do so, unless I had I had on gloves at the time.

"And lately even gloves do not seem to afford me entire protection. I know they are porous, and that, therefore, the subtle influence, whatever it may be, is capable of passing through them to my hands."

On my asking this lady whether she really believed in the theory she had constructed, she answered that at times she was convinced that she was in error, but only for a short period, as the original ideas returned in full force; that when reasoned with in regard to the absurdity of her notions, she was persuaded for the moment that she was wrong, but as soon as she was left to herself, she was back in the old train of thought.

The expression of the patient was one of anxiety. As she sat talking to me she was continually rubbing her hands together, and looking at them closely every moment. After I had felt her pulse, she took a handkerchief from her pocket, moistened it with a little cologne water, which she had in a vial, and wiped the spot which my fingers had touched.

To test the sensibility of the hands, I made use of the æsthesiometer, without, however, detecting any abnormal condition, but she at once took another handkerchief, and wiped all the places touched, with cologne as before. She had a pocket full of clean handkerchiefs, never using the same one twice, and putting the soiled ones in another pocket.

She has given up reading, because handling books or newspapers was, she was sure, a certain source of pollution. At first this idea was applied only to books from a library to which she was a subscriber, but latterly it had been extended to all printed matter.

Further examination showed that she was subject to almost continual headache, mainly at the vertex, and that she had occasional attacks of dizziness. She slept badly, frequently getting only two or three hours of disturbed slumber. Her pulse was 96, weak and irregular. The heart-sounds were normal, but the action of the heart was feeble, with an irregular rhythm, and an occasional intermittence. The ophthalmoscope showed nothing abnormal, beyond a possible slight increase in the red tinge of the disk.

Menstruation was regular in every respect, but there was decided gastric dyspepsia. The phosphates of the urine were in great excess; in other respects there was no derangement of this excretion.

No hereditary tendency to insanity or other neurotic condition existed. Previous to the occurrence of the mental disorder in question she had been of equable temperament, and not at all disposed to melancholy or depression of spirits. Now, however, her mind was filled with the most gloomy forebodings and apprehensions. Her life was one continued state of fear lest she had become contaminated by something she had touched, and had forgotten to wash her hands after the contact, or had imperfectly washed them.

So far as could be discovered, there was no existing source of trouble or anxiety. She was in affluent circumstances, and had lived happily with her husband, who had, however, died a year after marriage of phthisis, with which he had been affected several years. There had been no pregnancy.

CASE IX.—Miss F——, aged 18, tall and slender, consulted me January 23d of the present year. From herself and her mother I obtained the following history:

About eighteen months previously she had gone to stay in the country with some friends, and on one occasion slept in a farm-house. On her return home she at once took a bath, and had her head, the hair of which was very long and thick, thoroughly washed; to her great surprise and disgust, it was found to be full of lice. She had always been exceedingly cleanly as regarded her person, and the shock she experienced on learning of the presence of these parasites completely unnerved her. She insisted on repeated washings of the head with soap, carbolic acid and other detergent and disinfectant substances, and even then was not convinced that all the vermin had been destroyed.

This was the starting point of all the subsequent mental disturbance. Little by little the idea became rooted that she could not escape sources of contamination, that other persons might defile her in some way or other, and that the various articles about her might also possess a like power. She was particularly careful in regard to avoiding children, and would not on any account allow a child to touch or even to approach her closely. When she went out into the street she carefully gathered her skirts together on passing any person, for fear that she might by mere contact be contaminated. She spent hours every day in minutely examining and cleansing her combs and brushes, and was even then not satisfied that they were thoroughly purified.

As to her hands, she washed them, as her mother informed me she had ascertained by actual count, over two hundred times a day. She could touch nothing without feeling irresistibly impelled to scrub them with soap and water. Gradually the idea of lice had been lost sight of, and for several months previously to her coming to me, the fear of pollution had had a much more extended source. She could not define with any exactness what the *materies pollutionis* was, though she imagined it to be something that was capable of doing her bodily injury in some subtle manner by being absorbed into her system through her hands or other parts.

Some little time before coming under my observation she had extended her fear of contamination to the soap with which she felt compelled to wash her hands, and hence she was obliged to wash them again in pure water in order to remove all traces of the soap. Then, as the towel with which she wiped them dry had been washed with soap, she rinsed her hands in water, and allowed them to dry without the aid of a towel.

In removing her clothes at night preparatory to going to bed, she carefully avoids touching them with her hands, because then she would not have sufficient opportunity for washing. She, therefore, has some one else to loosen the fastenings, and then allows her garments to drop on the floor, where she leaves them. Nothing would persuade her to touch any of her under-clothing after it has been worn till it has been washed. A great source of anxiety with her is the fact that her clothes are washed in the laundry with the clothing of other people, but she sees no practicable way of escape from this circumstance. It none the less, however, makes her very unhappy.

When not washing her hands or examining her combs and brushes, she spends nearly all the rest of the day in carefully inspecting every article of furniture, and dusting it many times.

Thus her whole life is one continued round of trouble, anxiety and fear. Her whole character and disposition have changed. She is suspicious of every person and everything.

She is subject to insomnia, frequent headaches, and loss of appetite. There are noises in her ears, flashes of light before the eyes, and an utter impossibility of concentrating the attention upon any other subject than the one which has obtained so complete a mastery over her. Her menstruation is scanty and somewhat painful, though regular in other respects.

Ophthalmoscopic examination showed the retinal vessels to be increased in size, and the choroid to be of a deeper hue than is ordinarily met with.

Upon conversing with this young lady I had no difficulty in getting her to admit the absurdity of her ideas. She stated that whenever she reflected upon the subject she was convinced of their erroneous character, but that nevertheless she could not avoid acting as she did, for as soon as she was exposed to any possible source of contamination, the ideas returned in full force. It was only when she had, as she thought, done her best to cleanse her hands, that she doubted the correctness of her notions which had so thoroughly become a part of her mentality.

Case X.—Mrs. R. H—— came to my clinique at the University Medical College, and in my absence was recorded by Dr. Morton, as having delusions relative to filth or poisoning, to which she believed herself to be exposed. I knew nothing of this case till, on reading to him the foregoing statement of the chief symptoms I had observed, and which I told him, in my opinion, constituted a distinct type of mental aberration, he informed me of Mrs. H.'s case.

I saw her first on the 6th of March of the present year, and I have persuaded her to come here this evening, in order that the members may have an opportunity of examining a case of the interesting form of mental disorder under consideration.

From her and her husband I gather the following points in her clinical history:

About four months since, a window out of which she was looking, fell on her head, giving her quite a severe blow, from the effects of which she suffered for several days. A week or two after this event, she noticed that a brass candlestick was covered with verdigris, and she undertook to clean it. The fact made a great impression on her, and she at once looked up all the brass articles in her household and gave them a good scrubbing. She then conceived the idea that so much handling of brass would be injurious to her, so she began the hand-washing, which she continues up to the present time, not only after touching brass, but any other thing to which she is not thoroughly accustomed. I had her come to my house in order that I might examine her more thoroughly, but she would not touch the bronze bell-handle, or any of the door-knobs, for fear of contamination. Her husband was with her, and as she was going out of my consulting-room, I watched her to see how she would manage to get out. She would not touch the knob; nothing, she said, would persuade her to do so; it was impossible; so she stood the picture of fear till her husband opened the door for her.

She has also an extraordinary fear of handling coin, especially copper and silver, and experiences great distress after contact with paper money. She thinks it strange that other people can touch all these things without fear of defilement.

Unlike the other cases, she is impressed with doubts in regard to the propriety or advisability of her actions. Is uncertain how much salt to put into her food, is distrustful in regard to the quantity of coffee or water to use in preparing her breakfast. Is afraid the meat is being over-cooked, though only just put on the fire; if she has anything in her hand, is doubtful where to put it. For instance in ironing her clothes, she will hold the iron in her hand uncertain whether she ought to put it on this or that part of the table. Is never sure that her hands are clean, and washes them again and again. In washing and dressing her baby she is thoroughly confused relative to the putting on of its clothes; shall the stockings go on before the shirt, or shall the shirt go on first? Shall she wash its face before washing its hands? and so on, so that a duty which used to take only half an hour for its performance, now requires a whole morning.

If, however, she is told by one in whom she has confidence or whose authority she respects, what to do, she has no difficulty in performing any act about which she would otherwise be doubtful.

She has the *folie du doute*, of Le Grand du Saulle, but, differently from his cases, this condition was not first in order of occurrence.

It appears to be impossible for her to keep her hands still. She is always rubbing them together, interlocking the fingers or closing and opening them alternately.

She suffers a great deal from pain in the head and dizziness, and does not sleep well. Her digestion is deranged; she is constipated; her pulse is quick and irregular. There is no derangement of the menstrual function.

The constant apprehension that she labors under that she will touch something, the contamination of which cannot be removed by washing, or which she will overlook, makes her excedingly unhappy. It forms the subject of her conversation, causes her to neglect her household duties, and hence interferes very materially with the comfort and well-being of her household and children. She has become timid, and is, hence, afraid to go out alone or to undertake any work. While in the street she is never certain of her way, and is in continual terror that something capable of polluting her should, without her knowledge, come in contact with her. When I reason with her in regard to the incorrectness of her ideas, she at once admits their falsity, but only for the moment, for the very instant after telling me that she knew she was wrong, she could not bring herself to the point of touching the bronze knob on the door of my consulting-room.

These cases sufficiently show the chief characteristics of this variety of mental aberration, which I have ventured to call Mysophobia. Of course it is not contended that it is a distinct pathological entity. It is simply one of a group of which pyromania, kleptomania, entomania, etc., are members. Neither is it a fully developed form of insanity, for the subjects have a certain amount of control over its manifestations, and are conscious of the erroneous nature of their ideas.

Nor do I think it is identical with the *folie du doute* (*avec délire du toucher*) of Le Grande du Saulle, as the following description, which I take from his monograph on the subject, shows:

"The first period, which is entirely compatible with physical and mental health, consists of the spontaneous, involuntary and irresistible production of a certain series of theoretical, abstract and ridiculous thoughts on various subjects, without illusions or hallucinations. This series of thoughts may be expressed by notes of interrogation to questions continually addressed by the patient to himself, put with a feeling of profound but vague doubt, but at the same time with a degree of deliberation which is monotonous, overpowering and persistent. Again, there is a mental representation of certain things and reflections in regard to them which are more or less fixed in character. The internal struggle is a silent one. The subject never complains of the contest which is going on within him.

"The second period is manifested by the following phenomena: Unexpected revelations to the family, the friends and others about him; exaggerated scruples; chimerical fears; apprehensions and distress; periods of excitement with precedent epigastric aura; aversions to some animals; appreciable diminution of the idea of doubt and of the personal questionings; a morbid tendency to repeat the same things to the same person, and of being re-assured of any circumstance in exactly the same terms as were previously used; fear of touching certain objects; abnormal instincts of propriety; repeated washings; multiplied eccentricities; spontaneous avowal of the performance of ridiculous acts; long-continued intermissions in the course of the symptons still possible; complete preservation of the entire intelligence.

"The third stage is characterized by the presence of a permanent and seriously morbid state. The condition becomes every day more intolerable; all feeling of sensibility disappears; many of the normal actions of life become impossible; going out of the house is extremely repugnant, and at last is given up altogether; the actions become slower and slower, and many hours are passed in dressing in the morning and in eating the meals of the day; the circle of delirious ideas beçomes narrowed, and the mental distress augments in proportion; the fear of walking, sitting down, of coming in contact with any one, of shaking hands, of opening a window or a door, and unconquerable aversions for one object and another increase; the feeling of terror is no longer expressed in words, and the movements of the lips alone indicate the persistence of a mental language; complete consciousness of the situation persists; dementia never ensues, and life is prolonged indefinitely."

It will be evident from this outline description that the cases I have cited are not such as form the basis of M. Le Grand Du Saulle's *folie du doute* (*avec délire du toucher*). Some of us, doubtless, have witnessed such instances as he mentions. I can recall several which have occurred within the course of my own experience. It will be seen that the fear of touching certain objects, and the washing of the hands, to which he refers, are only incidental phenomena closely connected with the feeling of doubt which fills the mind of the patient, and have no relation to the fear of contamination which constitutes so prominent a feature of the cases detailed in this memoir.

Mysophobia is certainly closely allied with various hypochondriacal and hysterical forms of disease. Perhaps the nearest analogue is the sexual hypochondriasis or spermatophobia, of which all of us have seen many cases, and in which the miserable subject is continually haunted with the idea of impotence, softening of the brain, paralysis, etc., merely because he has an occasional nocturnal emission, or mistakes the escape of a little urethral mucus for the passage of semen. I have never, however, seen a case in a male, and though probably there are such, I am sure it is much more common with women than with men.

In several of my cases the affection had lasted two or three years before coming under treatment, and yet there did not appear to be any tendency to development into a more advanced type of mental derangement. The disorder appears to reach its full development in the course of a few months.

At no time is the intelligence of the patient weakened; even when the affection is at its height, or during the existence of a severe exacerbation, she is able to recognize the absurdity of the ideas which overpower her, and to resolve that she will endeavor to combat them; and although not successful in this contest, she nevertheless returns to the struggle day after day.

I have treated all my cases upon one general plan, and this has been so successful that I see no occasion for departing from it. As there is usually a tendency to constipation, I administer every alternate night a pill composed of 0.2 gram of podophyllin and 0.20 gram each of extract of aloes and inspissated ox-gall. Indeed, even were there no constipation I would use this pill, with the object of acting thoroughly on the liver and intestines. The intention is not to violently purge the patient, however, and therefore if it acts too powerfully, one of less quantities should be administered.

In addition I give the bromide of sodium or potassium or calcium, to such an extent as to bring the patient quickly and completely under its influence, and if there is a decided tendency to melancholia I give opium in combination.

It is not long before amelioration begins; the patient's mental strength improves day by day, and she is the better able to contend with the ridiculous notions which govern her. The periods during which she is entirely convinced of her errors become longer and more frequent, and the false ideas themselves are less vivid. In the course of three or four months at the utmost, the patient is, according to my experience, free from mental aberration, though greatly reduced in strength. This, however, is not a matter of much consequence. The stoppage of the medication, and the administration of small doses of strychnia, iron and quinine, with cod-liver oil and a full, generous diet, will complete the cure. All the cases under my care recovered, with the exception of the two now under treatment, and the result with them, as I judge by the steady improvement they have undergone, is merely a matter of time.

